# Annotations

**Published:** 1899-08-12

**Authors:** 


### ANNOTATIONS.

### Dr. Thresh on the Essex Water Supply.

As the result of an investigation which he has made regarding the character of the water furnished by the wells in Essex, Dr. Thresh, medical officer of health for that county, is of opinion that the continued pumping from the deep wells which has been going on for some time has lowered the water level to such an extent below the ordnance datum as to lead to a flow of sea water into the chalk strata. It appears that the chalk underlies the whole of Essex, one outcrop being at the west and the other to the east, the latter being under the sea. The vast underground reservoir formed by the chalk strata tends, then, to be supplied from two sources —the rain which falls upon the land outcrop to the west, and the sea water which covers the outcrop to the east. Under natural circumstances, the land outcrop being the highest, the tendency is for a constant but very slow percolation of rain water to pass through the chalk eastwards towards the sea, thus keeping out any fresh incursion of sea water. But directly the subterranean water level is lowered, as has now been done, this current is reversed, sea water flows in, and the wells tend to become brackish. This is what has already happened. Observation of the character of the water in various parts of the county, and of the changes which it has undergone, leads Dr. Thresh to the opinion that depletion of the great underground reservoir of water in the chalk underlying the county of Essex is far more likely to be replaced by sea water than by rain water; and, in fact, that very little of the water now being removed is being replaced from the rainfall on the land outcrop on the west. On both south and east wells sunk into the chalk have had to be abandoned on account of the brackish character of the water obtained, and Dr. Thresh is of opinion that the over-pumping from the chalk in the south of Essex, and the continued depression of the water-level below that of the sea, will lead to infiltration of sea water on the east and of tidal water on the north and south.

### School Space.

A correspondent in the *Pall Mall Gazette* draws attention to the very large expenditure caused by the accommodation in the schools of the London School Board being calculated on the basis of 10 square feet for each child instead of the eight square feet required by the Education Department. His object in so doing has relation to the perennial conflict between the voluntary and the Board schools, with which we have nothing to do. The matter, however, is one of very considerable interest in regard to questions of health, and when we see the very large expenditure which is involved in providing even only a couple of feet more floor space per child than the voluntary schools have been accustomed to give, it becomes a serious question whether the introduction of mechanical aids to ventilation, to a very much larger extent than is common at the present time, would not be of greater advantage, both in regard to economy and efficiency, than the enlargement of the floor space and cubic space which has been going on of recent years. The fact is that the schools have already reached, even if they have not already exceeded, the limit of floor space which is practically permissible on educational grounds, unless the number of scholars in each class is to be considerably diminished. It may be different in the case of older students—although even with them it is an immense advantage to be near the teacher—but with children it is an absolute necessity that they should be kept together well under the teacher's eye. If the teachers had their own way there can be no doubt that in many cases, although there might be a full average of 10 feet per child take the room altogether, each child would not have its own ten feet. They would in fact be huddled together as near the blackboard as possible, and the rest of the room would be empty. We quite recognise that the ventilation of many of the modern schools is very much better than in the older ones, but there are reasons which make us doubt whether we have yet arrived at anything like the best possible method for ensuring pure air for children while at school. Mechanical ventilation has in the minds of many people been far too much bound up with what is called the "plenum system." But other methods are available, and we think it is worthy of serious consideration whether, in some of the schools to be erected by our great school boards, some attempt should not be made (experimental though it might be) to ventilate the class rooms on the same principles as have been found effectual in the ventilation of the workshops in certain dangerous trades. In large towns, where land and buildings are frightfully expensive, such a plan ought not to be as expensive as providing extra space, while it certainly ought, for the particular purpose in hand, to be more effectual than what is called natural ventilation, which too often means getting a good blow through during the interval between the classes, and bearing the stuffiness as well as one can while the classes are going on.

### Trade Risks to Eyesight.

Few of us, perhaps, recognise the dangers to sight that are involved in some occupations. Mr. Simeon Snell, in his presidential address to the ophthalmological section at Portsmouth, said that in many trades associated with iron and steel small foreign bodies were very apt to be lodged in the workmen's corneæ. Even in the course of a single day a grinder might get several such bodies fixed in his cornea. If the cornea of a grinder were carefully examined with a magnifying glass it would not infrequently be found to be studded over with minute nebulæ caused by the repeated slight injuries which had been thus received, and, if further testimony of the risks to which the grinder's eyes are exposed were required, it might be found by examining the spectacles of such grinders as use them. The surface would be found to be studded all over with marks caused by the impact of particles of steel or emery. Many workmen were, he said, very skilful in removing "motes," as these particles are called when they stick in the cornea, and the number which they removed in the course of a single day was sometimes very large. One man, a timekeeper at works where 1,000 men besides outworkers were employed, stated that sometimes he had extracted a score or more a day, sometimes less, but that for many years he had not passed a day without having had at least one case. He, however, was not the only man at these works who had a reputation for removing "motes," and, probably, the total number of such accidents was very large indeed.

